# Treadmill Exercise Decreases Aβ Deposition and Counteracts Cognitive Decline in APP/PS1 Mice, Possibly *via* Hippocampal Microglia Modifications

**DOI:** 10.3389/fnagi.2019.00078

**Published:** 2019-04-05

**Authors:** Xianliang Zhang, Qiang He, Tao Huang, Na Zhao, Fei Liang, Bo Xu, Xianghe Chen, Tuojian Li, Jianzhong Bi

**Affiliations:** ^1^Shandong University, Jinan, China; ^2^College of Physical Education, Shandong Normal University, Jinan, China; ^3^Department of Physical Education, Shanghai Jiao Tong University, Shanghai, China; ^4^School of Physical Education & Health Care, East China Normal University, Shanghai, China; ^5^College of Physical Education, Yangzhou University, Yangzhou, China; ^6^Department of Neurology Medicine, Shandong University, Jinan, China

**Keywords:** Alzheimer’s disease, treadmill exercise, cognitive function, amyloid-β, microglia, neuroinflammation, oxidative stress

## Abstract

Recent studies have suggested that exercise may be beneficial for delaying or attenuating Alzheimer’s disease (AD). However, the underlying mechanisms were not clear. Microglia-mediated neuroinflammation is suggested to play an important role in the pathology of AD. The present study investigated the beneficial effects of treadmill exercise on amyloid-β (Aβ) deposition and cognitive function in amyloid precursor protein (APP)/PS1 mice in the early stage of AD progression and microglia-mediated neuroinflammation was mainly analyzed. The results demonstrated that 12 weeks of treadmill exercise preserved hippocampal cognitive function in APP/PS1 mice and substantially suppressed Aβ accumulation in the hippocampus. Treadmill exercise significantly inhibited neuroinflammation, which was characterized by a remarkably reduced expression of pro-inflammatory factors and increased expression of anti-inflammatory mediators in the hippocampus, resulting from a shift in activated microglia from the M1 to M2 phenotype. Treadmill exercise also attenuated oxidative stress presented by a marked reduction in methane dicarboxylic aldehyde (MDA) level and dramatically elevated SOD and Mn-SOD activities in the hippocampus. These findings suggest that treadmill exercise can effectively prevent the decrease in hippocampal-dependent cognitive function and Aβ deposits in early AD progression possibly *via* modulating microglia-mediated neuroinflammation and oxidative stress.

## Introduction

The process of aging is accompanied by various health problems that are highly challenging to public medical and health systems. Dementia is a common health problem in elderly people. The most common form of dementia in elderly people is Alzheimer’s disease (AD), which may lead to irreversible cognitive deficits (Huber et al., 2018). Amyloid-β (Aβ) deposition and neurofibrillary tangles are two established hallmarks of AD linked with AD pathology. Previous studies indicated that neuroinflammation may be the third disease component and has recently received much attention (Ardura-Fabregat et al., 2017). It’s believed that there are a large number of microglia around the Aβ plaques (Cui et al., 2016). Excessive activation of microglia release numbers of pro-inflammatory factors, such as interleukin-1 (IL-1), tumor necrosis factor-α (TNF-α), thereby promoting amyloid precursor protein (APP) expression and Aβ deposition (Lian et al., 2016), suggesting that microglia-mediated neuro-inflammatory response plays an important role in Aβ deposition and the development of AD. In fact, Heneka et al. (2013) reported that chronic neuroinflammation emerged in the brain before the deposition of Aβ. Unfortunately, there are no specific treatments or effective drugs that can reverse the progression of AD (Hickman et al., 2016). However, exercise, a modifiable lifestyle can reverse cognitive impairment, and slow the progression of AD (Liu et al., 2013; Marlatt et al., 2013; Zhao et al., 2015; Hüttenrauch et al., 2016; Koo et al., 2017). Furthermore, numerous studies have suggested that microglia regulation may be a key mechanism of the beneficial effects exerted by physical exercise (Xiong et al., 2015; Elahi et al., 2016). However, one study did not find that the activation of microglia was affected by exercise interventions in AD (Zhang et al., 2018). Therefore, the effect of exercise on the microglia of AD is not clear yet.

Microglia are considered the resident immune cells in the brain. When microglia are activated, they phagocytose invading components and release cytokines. In the case of AD, microglia fail to carry out their normal functions, such as clearance of pathological protein aggregates and cell debris. They become chronically activated, continuously releasing neurotoxic substances, contributing to the pathogenesis of AD. In mouse models such as APP/PS-1, activated microglia accumulate in the vicinity of Aβ plaques; upregulation of microglial markers has been reported in these mice as early as 3 months and persists at 14 months and 24 months (Kelly, 2018). Activation of microglia exhibit diverse phenotypes (M1 and M2), which can result in two opposing effects (Wilcock, 2012). M2 phenotype microglia exert beneficial anti-inflammatory effects leading to the clearance of Aβ and the release of neurotrophic factors; M1 exert detrimental effects caused by the release of pro-inflammatory cytokines and free radicals (Tang and Le, 2016). Several recent studies focused on the shifting of microglia from an M1 state to an M2 state, and this shift reduces the toxic effects and enhances Aβ clearance (Tang and Le, 2016). In the progressive pathological process of AD, a gradual increase of activated microglia indicated a shift of M2 microglia toward M1 microglia which was accompanied with increased expression of pro-inflammatory cytokines and chemokines (Hoozemans et al., 2006). Meanwhile, it was also reported a shift of M2 microglia toward M1 microglia in the hippocampus of aged APP/PS1 AD mice (Zhang and He, 2017). An animal study showed that the expression of IFN-γ in hippocampus increased gradually with age which induced the activation of microglia, while the expression of anti-inflammatory IL-4 decreased (Nolan et al., 2005). Therefore, microglia present different phenotypes in brain in different stage of AD. It was previously reported that amyloid plaque deposits first start to appear at the age of 6 months in APP/PS1 mice (Zhang et al., 2012). Therefore, 3-month-old APP/PS1 mice were used to investigate the effects of 3-month exercise on microglia phenotype of the initial stage of AD, aiming to provide a reference for the prevention effect of exercise on AD.

The present study investigated the effects of treadmill exercise on microglia-mediated neuroinflammation in the hippocampus of APP/PS1 mice. The results revealed that treadmill exercise significantly suppressed neuroinflammation and inhibited oxidative stress in the hippocampus of AD animal models. Notably, treadmill exercise increased the number of M2 phenotype microglia, with a corresponding suppression of the M1 phenotype microglia in the hippocampus of AD. These effects were associated with profound improved cognitive function and reduction of Aβ deposition.

## Materials and Methods

### Animals

Heterozygous APPswe/PS1De9 (APP/PS1) double-transgenic mice with a C57BL/6 background were used in the present study. The mice were obtained from the Model Animal Research Center of Nanjing University (Nanjing, China). Three-month-old male APP/PS1 mice were randomly divided into a transgenic control group (TC) and transgenic exercise group (TE). C57BL/6 mice were used as the wild-type control group (WC). Each group consisted of six mice. All mice were housed individually in standard plastic cages under conventional laboratory conditions (22–24°C, 40%–60% relative humidity) with *ad libitum* access to food and water and maintained on a standard 12/12-h light/dark-cycle with lights on at 6:00 am. The Experimental Animal Care and Use Committee at East China Normal University approved all experimental procedures (license number for the use of laboratory animals), which conformed to the guidelines for the use of laboratory animals published by the People’s Republic of China Ministry of Health. All efforts were made to minimize the number and suffering of animals in these experiments.

### Treadmill Exercise Protocol

The treadmill exercise training protocol was adapted from previous studies (Um et al., 2011; Liu et al., 2013). Mice in the TE group received a 6-day familiarization period to adapt to their new environment followed by 12 weeks of exercise training. The familiarization period (15 min/day) was designed for the mice to learn how to run on a horizontal treadmill (Xinruan Technology CO., Ltd, Hangzhou, China). The treadmill speed on the first 2 days (days 1–2) was 5 m/min, and increased to 8 m/min on days 3–4. The final speed was 12 m/min on days 5–6. The mice were subjected to the exercise protocol from 6 to 8 p.m., for 45 min/day, 5 days/week for 12 weeks. Mice performed the following speed sequence each day: 5 m/min for 5 min, 8 m/min for 5 min, 12 m/min for 30 min, and 5 m/min for 5 min. Mice in the WC and TC groups were placed on a static treadmill for the same amounts of time.

### Morris Water Maze Task

The Morris water maze was used to assess spatial learning and memory performance. The detailed method was described in a previous report (Zhang and He, 2017). In brief, mice were trained to swim to the platform in a pool for five consecutive days in the navigation experiments. Training consisted of three 1-min trials per day. Average escape latency and percent of time in the platform quadrant to find the submerged platform was obtained and averaged for each day of acquisition. A spatial exploration experiment, which consisted of 1 min of free swimming in the pool without the platform, was performed on day 6. The number of crossing times and distance percent in the platform quadrant were determined from video tracking records.

### Novel Object Recognition Test

The novel object recognition test was used to evaluate recognition memory performance. The detailed method was described in a previous study (Cohen et al., 2013). The test was divided into 3 days. Mice were allowed 5 min to explore an empty recognition box for habituation on day 1 (adaptive period). Mice were placed in the same recognition box on day 2 (training period) with two identical objects at an equal distance and given 5 min to explore. Mice were returned to the same recognition box with a familiar object and a novel object on day 3 (testing period). The novel object was different in shape and color and consistent in height. Exploration of the object was defined as the animal’s nose being within 2 cm from the object. The times spent exploring the familiar and novel objects were recorded. The discrimination index was calculated as the time spent exploring the novel object/the time spent exploring novel object + the time spent exploring a familiar object.

### Immunohistochemistry

As described in a previous study (Liu et al., 2013), immunohistochemistry was performed to analyze the distribution of Aβ plaques. Briefly, paraffin sections were deparaffinized with xylene, rehydrated in a graded serious of alcohol solutions, and then treated with 0.1 M Tris-HCl-buffered saline (pH 7.4) containing 3% (H_2_O_2_) for 10 min to reduce endogenous peroxidase activity. Sections were then incubated overnight with a mouse anti-Aβ antibody (MABN10, Sigma; 1:100) at 4°C. After rinsing, sections were incubated with biotinylated goat anti-mouse IgG (1:200) for 1 h. After rinsing, sections were stained with 0.025% diaminobenzidine (DAB) for 5–10 min. Hematoxylin staining was performed for 8–10 min, and sections were rinsed. The stained sections were dehydrated through a series of graded alcohol solutions, cleared with xylene, and covered with neutral balsam. Sections were imaged under an upright microscope (Leica, Germany). The area of plaques was measured using the optical fractionator technique. The data were analyzed using Image-Pro Plus 6.0 software (Media Cybernetics, Rockville, MD, USA).

### Double Immunofluorescence Staining

In order to examine the relevance between Aβ and activated microglia in the hippocampus of APP/PS1 mice. A double immunofluorescence (IF) staining was carried out. In this study, we used mouse anti-Iba1 antibody (SAB2702364, Sigma) to label activated microglia, while rabbit anti-beta amyloid antibody (ab2539, Abcam) to identify amino acid residues 1–14 of Aβ and stain extracellular aggregates of Aβ peptides. Before the double IF staining, warm up slides at room temperature for 30 min, and then fix the samples in 4% paraformaldehyde in PBS pH 7.2–7.4 for 15 min at room temperature, and wash the samples three times with ice-cold 0.01 M PBS, 5 min per time. After fixation, incubate the samples for 5 min with 70% formic acid and wash the samples three times with ice-cold 0.01 M PBS, 5 min per time. Incubate the samples with 3% BSA for 30 min to block unspecific binding of the antibodies, and then incubate the samples in the mixture of two primary antibodies (mouse anti-Iba1 antibody, 1:100; rabbit anti-beta amyloid antibody, 1:200) in 1% BSA in a humidified chamber for overnight at 4°C. Decant the mixture solution and wash the samples three times with ice-cold 0.01 M PBS, 5 min per time. Incubate the samples with the mixture of two secondary antibodies with two different fluorochromes (FITC-conjugated goat anti-mouse IgG, 1:200; rhodamine-conjugated donkey anti-rabbit IgG, 1:200) in 1% BSA for 1 h at room temperature in dark. Decant the mixture of the secondary antibody solution and wash the samples three times with ice-cold 0.01 M PBS for 5 min each in dark. Incubate the samples with 5 μg/ml DAPI for 10 min, and rinse with PBS. Mount coverslip with a drop of 50% glycerinum, movement under a microscope and take pictures. Double-stained brain samples were photographed using a fluorescence microscope.

### Flow Cytometry

CD11b^+^CD45^+^cells, CD11b^+^CD86^+^cells and CD11b^+^CD206^+^cells were assessed as activated microglia and M1 and M2 phenotype microglia, respectively, using flow cytometry according to a previous study protocol (Nikodemova and Watters, 2012). In brief, the hippocampus was homogenized in buffer A (1× DPBS + 0.5% BSA), and the homogenate was passed through a 70-μm nylon mesh cell strainer into a 50-ml conical tube containing papain buffer (PIPES + EDTA + L-cysteine-HCL + 80 U/ml DNaseI + 8 U/ml papain). Samples were centrifuged in a refrigerated centrifuge at 1,000× *g* for 15 min at 4°C. Supernatants were aspirated, and cells were resuspended in FACS buffer (1% BSA + 2 mM EDTA + 1× DPBS). Samples were centrifuged in a refrigerated centrifuge at 1,000× *g* for 10 min at 4°C. Supernatants were aspirated, and cells were resuspended in 30% Percoll. Samples were centrifuged in a refrigerated centrifuge at 700× *g* for 10 min. Supernatants were aspirated, and cells were resuspended in FACS buffer. All cells were incubated with an Fc-Block antibody for 20 min to prevent nonspecific binding. All hippocampal cells were incubated with antibodies against CD11b-Pacific Blue, CD45-FITC, CD86-PerCP-Cy5-5 and CD206-APC for 20 min. Expression of surface antigens was analyzed using a flow cytometer (FACS CantoII, BD, USA).

### RT-PCR

The mRNA expression of inflammatory mediators was examined using RT-PCR as described previously (Oh et al., 2017). First, total RNA of the hippocampus was extracted using a Trizol protocol. RNA (1 μg) was reverse transcribed into cDNA. The following primer sequences were used for PCR: TNFα (F) 5′-CGA GTG ACA AGC CTG TAGCC-3′, (R) 5′-ACA AGG TAC AAC CCA TCGGC-3′; IL-1β (F) 5′-ACC ACC AGC TTT GAG TCAGC-3′, (R) 5′-ATA AGG GGC TCT TCC GGTGT-3′; IL-10 (F) 5′-TAA CTG CAC CCA CTT CCCAG-3′, (R) 5′-AAG GCT TGG CAA CCC AAGTA-3′; TGF-β (F) 5′-AGG GCT ACC ATG CCA ACTTC-3′, (R) 5′-CCA CGT AGT AGA CGA TGGGC-3′; and GAPDH (F) 5′-AAT GTG TCC GTC GTG GAT CTGA-3′, (R) 5′-AGT GTA GCC CAA GAT GCC CTTC-3′. Real-time PCR was performed on 2 μl cDNA using SYBR Green real-time PCR master mix (TOYOBO). Threshold cycle (Ct) values were recorded, and mRNA expression was calculated using 2^−ΔΔCt^.

### ELISA

Hippocampal samples were homogenized in PBS (pH 8.0) and centrifuged at 3,000 *g* for 1 h at 4°C to remove insoluble material. The supernatant fractions were placed in ELISA plates for measurement of Aβ40 (KHB3482; Invitrogen, Carlsbad, CA, USA) and Aβ42 (KHB3442; Invitrogen).

Hippocampal samples were homogenized in PBS (pH 8.0) and centrifuged at 1,600 *g* for 10 min at 4°C to remove insoluble material. The supernatant fractions were placed in ELISA plates for measurement of methane dicarboxylic aldehyde (MDA) content (Beyotime, SO131) and SOD and Mn-SOD activities (Beyotime, SO103).

### Statistical Analysis

The data are reported as mean ± SD. All statistical analysis were performed using GraphPad Prism software (version 5.0). One-way ANOVA and further LSD-T test were used to analyze data.

## Results

**Figure 1:**
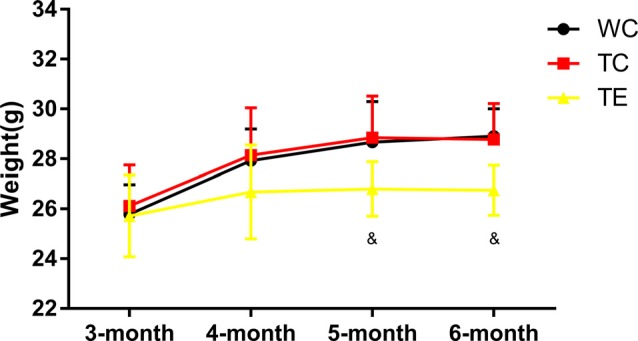
Effects of treadmill exercise on weight in amyloid precursor protein (APP)/PS1 mice. *n* = 6 per group. ^&^*p* < 0.05 vs. transgenic control (TC).

Animals in all groups showed an increase in weight with age. No difference was observed between TC group (28.77 ± 1.45 g) and WC group (28.91 ± 1.09 g) in weight; however, TE group (26.74 ± 1.00 g, *p* < 0.05) was significantly lower than TC group after 12 weeks of exercise intervention (Figure 1).

### Treadmill Exercise Improves Cognitive Function in APP/PS1 Mice in the Morris Water Maze Task and Novel Object Recognition Test

**Figure 2:**
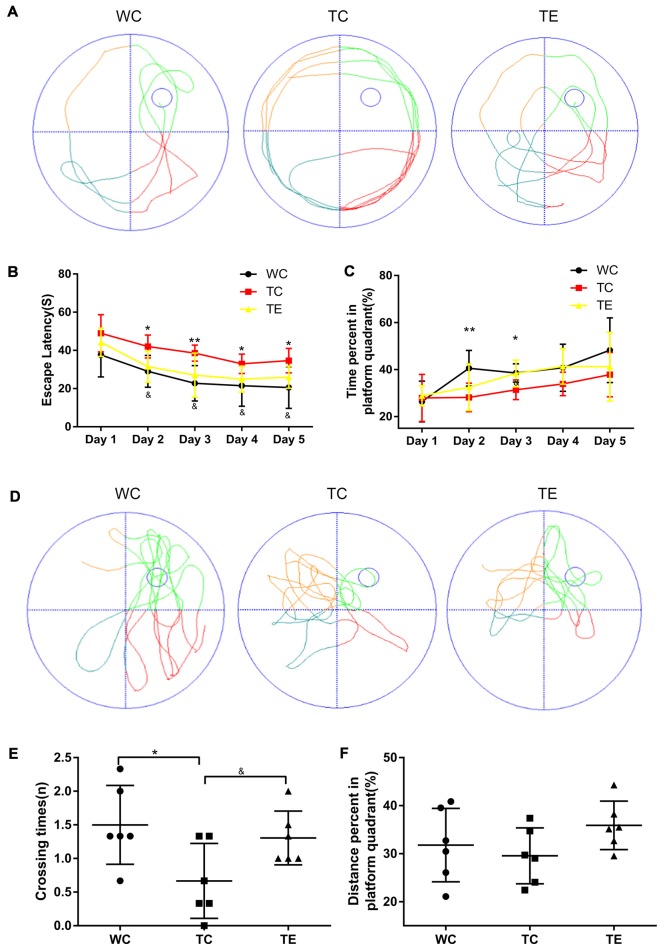
Effects of treadmill exercise on spatial learning and memory deficits in APP/PS1 mice. **(A)** Representative swimming trajectory of mice on the first trial day in WC, TC and transgenic exercise (TE) group. **(B)** Escape latencies of mice. **(C)** Percent of time spent in platform quadrant. **(D)** Representative swimming trajectories of mice on the sixth trial day in WC, TC and TE group. **(E)** Crossing times of mice. **(F)** Distance percent platform quadrant of mice. *n* = 6 per group. *p < 0.05 vs. WC; **p < 0.01 vs. WC; ^&^*p* < 0.05 vs. TC.

A hippocampus-dependent spatial learning and memory test, the Morris water maze task, was used to investigate the effects of 12 weeks of treadmill exercise training on spatial learning and memory functions of APP/PS1 mice (Cohen et al., 2013; Zhang et al., 2016). The TC group performed poorly in navigation tests and exhibited a longer escape latency on days 2–5 (42.01 ± 6.00; 38.59 ± 4.20; 33.00 ± 5.13; 34.67 ± 6.44) compared to the WC group (29.03 ± 8.35, *p* < 0.05; 22.74 ± 9.30, *p* < 0.01; 21.52 ± 10.73, *p* < 0.05; 20.60 ± 10.95, *p* < 0.05; Figures 2A–C). The TC also spent a shorter amount of time in the platform quadrant on days 2–3 (28.16 ± 6.09; 31.41 ± 4.21) compared to the WC group (40.56 ± 7.58, *p* < 0.01; 38.55 ± 3.92, *p* < 0.05). Notably, animals that underwent treadmill exercise exhibited remarkable cognitive improvement. TE mice exhibited a notable decrease in escape latency on days 2–5 (31.48 ± 8.23; 27.05 ± 11.55; 24.96 ± 6.80; 25.98 ± 5.82) compared to the TC group (42.01 ± 6.00, *p* < 0.05; 38.59 ± 4.20, *p* < 0.05; 33.00 ± 5.13, *p* < 0.05; 34.67 ± 6.44, *p* < 0.05) and a marked elevation in the percentage of platform quadrant occupancy on day 3 (38.30 ± 5.73) compared to the TC group (31.41 ± 4.21, *p* < 0.05). The platform was removed after the last training (5 days later), and the mice were given 1 min to find the missing platform in a probe trial. The number of times of platform crossings of the TC group (0.67 ± 0.56) was significantly less than the WC group (1.50 ± 0.59, *p* < 0.05; Figures 2D–F). However, higher numbers of platform crossings were observed in the TE group compared to the TC group (1.31 ± 0.40, *p* < 0.05). Notably, no significant differences in escape speed were noted between groups (data not shown).

**Figure 3:**
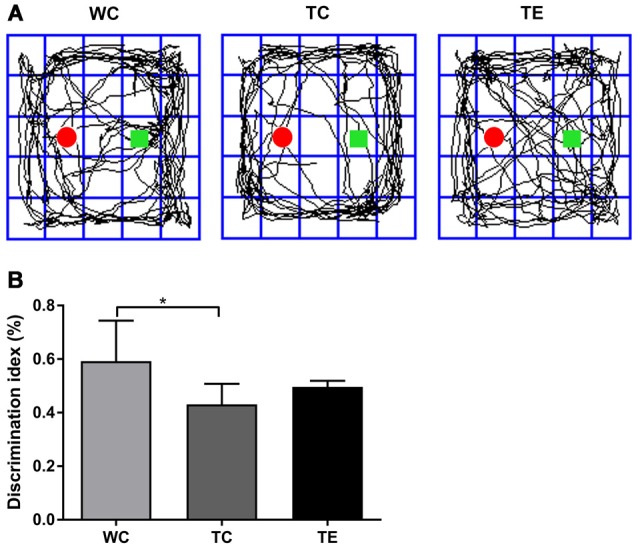
Effects of treadmill exercise on recognition memory deficits in APP/PS1 mice. **(A)** Representative traces are displayed for mice exploration of the familiar object and novel object in WC, TC and TE group. The red round circle marked the familiar object. The square marks the novel object. **(B)** Discrimination index of mice in each group. *n* = 6 per group. *p < 0.05 vs. WC.

A novel object recognition test was also performed to determine whether treadmill exercise preserved recognition memory in APP/PS1 mice (Gu et al., 2016). Recognition memory was impaired in TC mice, which exhibited a decreased preference to explore the novel object (0.43 ± 0.08) compared to the WC mice (0.59 ± 0.15, *p* < 0.05; Figure 3). However, the preference for novel object exploration exhibited an increasing rising trend in TE mice compared with the TC group (0.50 ± 0.02, *p* = 0.07).

### Treadmill Exercise Inhibits Elevations in Aβ Deposits in the Hippocampus of APP/PS1 Mice

**Figure 4:**
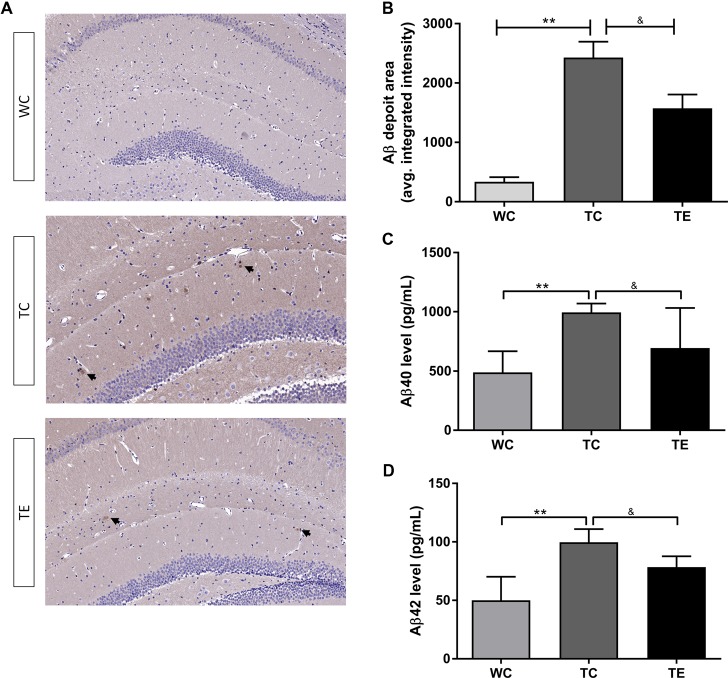
Effects of treadmill exercise on amyloid-β (Aβ) levels in hippocampi of APP/PS1 mice. **(A)** Immunohistochemistry staining of Aβ deposits in WC, TC and TE group. **(B)** Statistic analysis of Aβ deposit area in three groups. **(C)** ELISA of Aβ40 level in the hippocampi of WC, TC and TE group. **(D)** ELISA of Aβ42 level in the hippocampi of WC, TC and TE group. *n* = 6 per group. **p < 0.01 vs. WC; ^&^*p* < 0.05 vs. TC.

Aβ levels are upregulated early in AD progression, and this change strongly correlates with the decline in cognitive function (Pan et al., 2018). APP/PS1 mice develop Aβ deposits around 6 months of age and Aβ increase with age as previously reported. To determine if treadmill exercise can prevent the deposition of Aβ in the hippocampus of APP/PS1 mice in the early stage of AD progression, the Aβ load was indicated by average areas (Figure 4A). Clearly, in the hippocampus, Aβ load in the TC group (2427.50 ± 656.49) was much greater than the WC group (336.50 ± 189.26, *p* < 0.01) while a significant decrease in the TE group (1573.15 ± 30.572.49, *p* < 0.01) was observed when compared with the TC group (Figure 4B). We examined the levels of soluble forms of Aβ (Aβ40/42) and explored the Aβ plaque deposits in the hippocampus of all groups using Elisa and immunohistochemistry, respectively. Elisa showed that the generation of Aβ40 and Aβ42 in hippocampus was much higher in the TC group (993.87 ± 68.56; 99.74 ± 10.21) than the WC group (489.59.38 ± 162.45, *p* < 0.01; 26.90 ± 1.69, *p* < 0.01; Figures 4C,D), and a dramatic decrease was observed after 12 weeks of treadmill exercise (693.45 ± 309.10, *p* < 0.05; 78.49 ± 8.45, *p* < 0.05).

### Treadmill Exercise Shifts Microglial Polarization to the M2 Phenotype and Reduces the Production of Pro-inflammatory Cytokines

**Figure 5:**
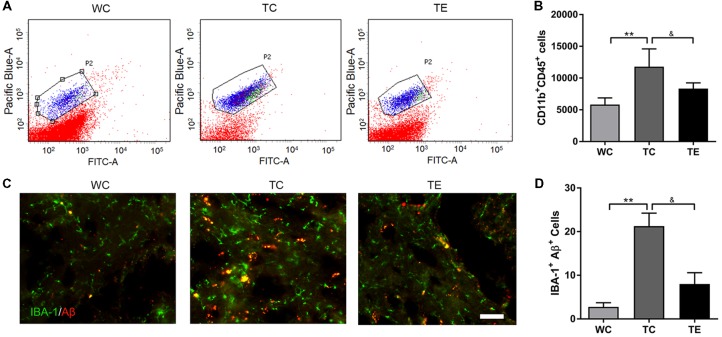
Effects of treadmill exercise on activated microglia in hippocampi of APP/PS1 mice. **(A)** Flow cytometry analysis of microglia. Pacific Blue-A and FITC-A represent CD11b and CD45, respectively. P2 represents CD11b^+^CD45^+^cells, which are activated microglia. **(B)** Quantitative analysis of CD11b^+^CD45^+^cells in three groups. **(C)** Immunofluorescence (IF) of microglia and Aβ in WC, TC and TE group, the Iba1 staining was performed to mark the microglia. **(D)** Statistic analysis of the number of microglia and Aβ protein double positive cells in each group. *n* = 6 per group. **p < 0.01 vs. WC; ^&^*p* < 0.05 vs. TC.

**Figure 6:**
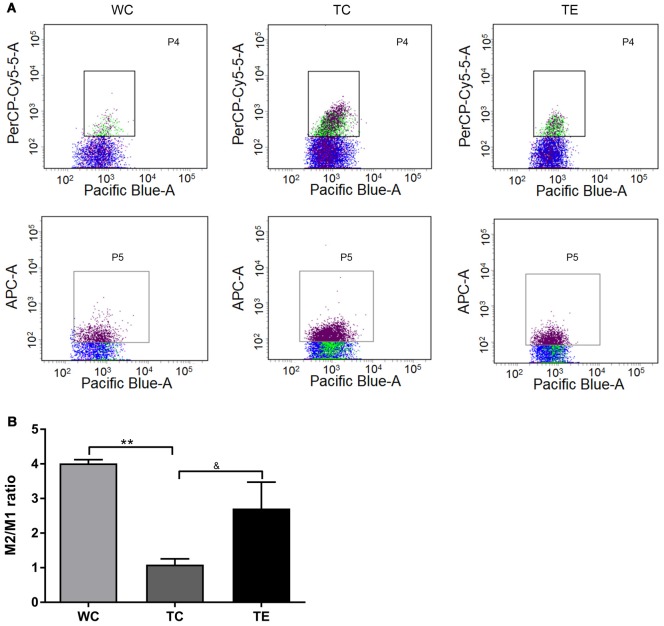
Effects of treadmill exercise on M1 and M2 phenotype microglia in hippocampi of APP/PS1 mice. **(A)** Flow cytometry analysis of M1/M2 microglia. Pacific Blue-A, PerCP-Cy5-5A and APC-A represent CD11b, CD86 and CD206, respectively. P4 represents CD11b^+^CD86^+^cells, which are M1 phenotype microglia. P5 represents CD11b^+^CD206+cells, which are M2 phenotype microglia. **(B)** Quantitative analyses of M2/M1 phenotype microglia of mice. *n* = 6 per group. **p < 0.01 vs. WC; ^&^*p* < 0.05 vs. TC.

**Figure 7:**
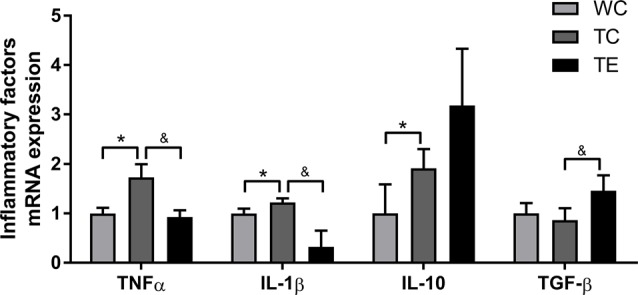
Effects of treadmill exercise on inflammatory factor mRNA expression in the hippocampi of APP/PS1 mice. *n* = 6 per group. *p < 0.05 vs. WC; ^&^*p* < 0.05 vs. TC.

Recent studies indicated that reactive gliosis plays an important role in AD pathology (Balducci and Forloni, 2018). Therefore, the effects of treadmill exercise on microgliosis and microglial polarization in the hippocampus of APP/PS1 mice were examined. Activated microglia were analyzed using two markers, CD11b and CD45, with flow cytometry (Babcock et al., 2015). The numbers of CD11b^+^CD45^+^cells were higher in the hippocampus of TC (11787.80 ± 2806.92) than WC mice (5828.00 ± 1048.22, *p* < 0.01; Figures 5A,B). A decrease in CD11b^+^CD45^+^cells was observed in the TE group (8336.00 ± 906.15, *p* < 0.05) compared to the TC group. To determine the activation of microglia, we co-stained microglia and Aβ by using Iba1 and Aβ antibody, respectively (Figure 5C). We showed that Aβ-associated microglia in the TC group (21.25 ± 2.98) is significantly higher than the WC group (2.75 ± 0.96). Moreover, Aβ-associated microglia are significantly decreased in the TE group (8.00 ± 2.58) vs. TC group (21.25 ± 2.98). Previous studies demonstrated that activated microglia were polarized to the divergent “classically activated” M1 “pro-inflammatory” or “selectively activated” M2 “repair-anti-inflammatory” phenotype and secreted pro- or anti-inflammatory cytokines in neurodegenerative disorders based on the particular *in vivo* microenvironment. CD86 is an M1 marker, and CD206 is an M2 marker (Zhou et al., 2017). Flow cytometry analysis was performed for M1 and M2 phenotypes to determine the effects of exercise on microglia polarization in the hippocampus of APP/PS1 mice. The TC group exhibited a significantly and strongly reduced ratio of M2 (CD11b^+^CD206^+^cells) and M1 (CD11b^+^CD86^+^cells) microglia (1.09 ± 0.17) compared to the WC group (4.01 ± 0.11; *p* < 0.01, Figure 6), suggesting that the activated microglia were polarized predominantly to an M1 phenotype in AD. In contrast, this decrease in the ratio of M2 and M1 was attenuated in the TE group (2.71 ± 0.76, *p* < 0.05, Figure 5) compared to the TC group, suggesting that treadmill exercise helped shift activated microglia to a potentially more beneficial M2 “repair/anti-inflammatory” phenotype. RT-PCR was used to measure the levels of specific pro-inflammatory cytokines (TNF-α, IL-1β) and anti-inflammatory cytokines (IL-10, TGF-β) in the hippocampus and confirm that the change in M1/M2 microglia phenotype following treadmill exercise actually correlated with a change in the production of pro- and anti-inflammatory cytokines. mRNA expression revealed an increase in pro-inflammatory cytokines TNF-α (1.50 ± 0.23) and IL-1β (1.38 ± 0.10) in the TC group compared to the WC group (0.87 ± 0.10, *p* < 0.05; 1.13 ± 0.11, *p* < 0.05, Figure 7), and a significant decrease in the anti-inflammatory cytokine IL-10 (1.01 ± 0.20) in TC compared to WC mice (0.53 ± 0.31, *p* < 0.05). However, treadmill exercise markedly reversed these effects. TE mice exhibited a notable decrease in the pro-inflammatory cytokines TNF-α (0.36 ± 0.37, *p* < 0.05) and IL-1β (1.69 ± 0.61, *p* < 0.05) compared to the TC group, and a marked elevation in anti-inflammatory cytokines (1.41 ± 0.30) compared to the TC group (0.83 ± 0.23, *p* < 0.05).

### Treadmill Exercise Ameliorates Oxidative Damage in the Hippocampus of APP/PS1 Mice

**Figure 8:**
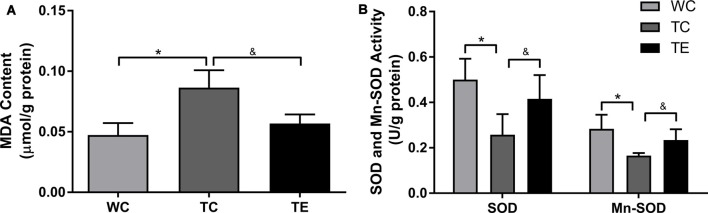
Effects of treadmill exercise on methane dicarboxylic aldehyde (MDA) content **(A)**, SOD and Mn-SOD activities **(B)** in hippocampi of APP/PS1 mice. *n* = 6 per group. *p < 0.05 vs. WC; ^&^*p* < 0.05 vs. TC.

Oxidative stress leads to oxidative damage of many cellular components in AD (Lee et al., 2018). Therefore, we examined the effects of treadmill exercise on oxidative damage in the hippocampi of APP/PS1 mice using MDA, SOD, and Mn-SOD. MDA content increased significantly in the TC group compared to the WC group (*p* < 0.05, Figure 8). Notably, treadmill exercise significantly decreased the content of MDA (*p* < 0.05). ELISA analysis revealed a decrease in SOD and Mn-SOD activities in the TC group compared to the WC group (*p* < 0.05), and treadmill exercise significantly reversed these effects (*p* < 0.05).

## Discussion

It is well established that exercise benefits overall brain health and cognitive function (Cotman and Engesser-Cesar, 2002). Consistent with other AD animal models (Adlard et al., 2005; Belarbi et al., 2011), we have previously reported that voluntary wheel running exercise improved cognitive deficits and attenuated Aβ deposits in APP/PS1 AD animal model. Recently, several studies showed treadmill exercise was also beneficial for AD pathology in APP/PS1 mice (Liu et al., 2011; Xiong et al., 2015). However, the underlying mechanisms are still not fully elucidated. The present study using an APP/PS1 AD animal model investigated the effects of treadmill exercise on Aβ deposits and cognitive function at the early stage of AD procession with microglia-mediated neuroinflammation mainly focused. Our findings demonstrated that 12 weeks of treadmill exercise produced significant neuroprotection of the hippocampus and preserved cognitive functions in the early stage of AD progression. Importantly, we demonstrated that treadmill exercise exerted neuroprotective effects by modulating microglia-related neuroinflammation in the early stage of AD progression, thereby attenuating the production and deposition of Aβ, and cognitive impairment.

In the present study, TC mice (6 months of age) were characterized by cognitive deficits in spatial learning and memory determined by the Morris water maze task and a novel object recognition test. The finding is consistent with a previous study in APP/PS1 mice (Trinchese et al., 2004). Aβ is the primary component of senile plaques, and its accumulation has long been proposed as a key event in facilitating the pathological progression of AD. Aβ weakens the structure and function of synapses, culminating in neurodegeneration and cognitive deficits (Cohen et al., 2013; Tapia-Rojas et al., 2016). Our results demonstrated a substantial increase in generation of Aβ and a higher Aβ deposition in the hippocampus of APP/PS1 mice, which is consistent with previous studies (Pedrós et al., 2014; Zhou et al., 2019). To determine whether treadmill exercise training can delay the progression of AD, the training was undertaken in mice from 3 to 6 months of age. The present study examined the effects of 12 weeks of treadmill exercise on cognitive function and hippocampal Aβ deposits. The results showed a significant cognitive improvement indicated by a notable decrease in escape latency on days 2–5, a marked elevation in the percentage of platform quadrant occupancy on day 3 and crossing times on day 6. In addition, 12 weeks of treadmill exercise also strongly inhibit the generation of Aβ and delayed the Aβ deposition in the hippocampus of APP/PS1 mice. And these finding is consistent with previous studies (Liu et al., 2013; Lin et al., 2015; Rao et al., 2015).

Proliferation and activation of microglia in the brain, concentrated around Aβ plaques, is a prominent feature of AD. Notably, the accumulation of Aβ in the hippocampus was associated with microglia activation and the induction of neuroinflammation in AD (Sarlus and Heneka, 2017). A previous study suggested that APP/PS1 mice at 10 months of age showed excessively microglia activation in the brain indicated by higher immune staining of microglia marker Iba1 (Xiong et al., 2015). In the present study, APP/PS1 mice at 6 months of age also presented a significant increase in activated microglia in the hippocampus using flow cytometry expressed as CD11b^+^CD45^+^ cells. This is also supported by an increased co-localization of Aβ in activated microglia. The present study also demonstrates that 12 weeks of treadmill exercise strongly reversed this increase, evidenced by a significant decrease of CD11b^+^CD45^+^ cells as well as Aβ-associated microglia in the hippocampus. However, Zhang et al. (2018) found that long-term treadmill exercise did not affect the activation of microglia in the hippocampus of APP/PS1 mice indicated by a co-staining of Iba1 and Aβ. The different results might be due to several reasons, including the time of exercise treatment and the exercise paradigm. In this study, 5-month-old APP/PS1 mice were used. It’s believed that a lot of AD symptoms have accumulated for 10-month-old APP/PS1 mice. Mice followed a running protocol of 30 min per day (5 m/min for 5 min, 8 m/min for 5 min, 11 m/min for 20 min) for 5 months and such training program might be not sufficient enough to reverse the microglia activation. And, similar results were observed in another study that 6 weeks aerobic voluntary running cannot counteract the AD pathology and the activation of microglia in 10-month-old APP/PS1 (Xu et al., 2013). However, Ke et al. (2011) used 7–8-month-old adult and 24-month-old aged APP/PS1 mice, and surprisingly 4 weeks of treadmill training clearly inhibited the activation of microglia in the hippocampus. In this study, the mice run at 10 m/min from 10 min/day to 60 min/day at the first week and maintained for 60 min/day for another 3 weeks. In addition, Xiong et al. (2015) used 4-month-old APP/PS1 mice and the results showed that 5 months of treadmill exercise did not attenuate the accumulation of Aβ, but attenuated the activation of microglia. In this study, the mice began with a distance of 70 m per day at a speed of 5–8 m/min, and gradually increased up to 300 m per day with a speed of 10–15 m/min in 1 month. Then the running distance was maintained constant until the end of the experiment. Therefore, the time of treatment and the volume of training every day might produce a different response. In our study, aiming to investigate the preventive role of exercise on AD, 3-month-old mice were used and received 3 months of exercise training. Thus, aerobic, if initiated at the early stage of AD, can be beneficial for preventing or delaying the progression of AD. On the contrary, if neurological health is compromised, aerobic exercise might not be effective to protect against AD pathology as several studies reported (Xu et al., 2013; Zhang et al., 2018). The preventive role of exercise may be more appreciated than the therapeutic effect for aged-related progressive disease. Meanwhile, our results are consistent with previous studies which used immunohistochemistry and suggested that treadmill exercise attenuated the increase in microglia activation in the brains of AD models to improve cognitive function (Ke et al., 2011; Xiong et al., 2015).

In addition, previous studies only show the changes of microglia numbers and failed to analyze the exact effect of exercise on microglia activity (Xu et al., 2013; Zhang et al., 2018). Microglia exhibit M1 and M2 phenotypes, and the present study found that a decreased ratio of anti-inflammatory M2/pro-inflammatory microglia in the hippocampus of APP/PS1 mice. One limitation of those studies which reported attenuation of activation of microglia by treadmill exercise was that they did not analyze the phenotype of microglia. In the present study, 12 weeks of treadmill exercise induced a potentially beneficial change in microglia polarization in APP/PS1 mice. The finding suggested that treadmill exercise effectively suppressed M1 phenotype microglia and enhanced M2 phenotype microglia in the hippocampus of APP/PS1 mice. A study on the effects of exercise in STZ-induced AD rats also found that treadmill exercise regulated microglia polarization to exert neuroprotection using M1 and M2 markers and Western blotting (Lu et al., 2017). However, the present study used flow cytometry to directly observe M1 and M2 microglia. The shift of microglia from an M1 to M2 phenotype following treadmill exercise was associated with parallel decreases and increases in the production of pro- and anti-inflammatory cytokines, respectively. The changes in the microglia-derived cytokine profile contributed to the decreased inflammatory damage and increased repair of neurons, which may partially underlie the overall beneficial effects of exercise on neurodegeneration and cognitive function. M2 phenotype microglia also exhibit a phagocytic function. Our study demonstrated reduced Aβ production after treadmill exercise, which may be associated with the enhancement of M2 phenotype microglia by exercise.

The present study also demonstrated that treadmill exercise powerfully suppressed oxidative stress and reduced oxidative damage to cellular components in the hippocampi of APP/PS1 mice. The anti-oxidative stress effects of treadmill exercise also likely contributed to the exercise-induced neuroprotection and cognitive improvement in the present study. The pathological role of oxidative stress in AD is well established, and oxidative stress is closely linked to the induction of neuroinflammation (Souza et al., 2013; Urrutia et al., 2014). Increased MDA content and impaired SOD activity were observed in AD, and these markers are sensitive indexes for the monitoring of cognitive alterations in AD progression (Pan et al., 2012; Wang et al., 2013; Xu et al., 2014). Notably, treadmill exercise effectively reversed the reduction of SOD and Mn-SOD activities and the increase in MDA content in the hippocampus of APP/PS1 mice. Several previous studies are consistent with our results.

Taken together, the results of our study demonstrated that treadmill exercise induced profound neuroprotective and cognitive-preserving effects in APP/PS1 mice in the early stage of AD progression. The beneficial effects of treadmill exercise were likely multifactorial, including but not limited to the inhibition of Aβ accumulation and suppression of oxidative stress and microglia-derived neuroinflammation. Our findings provide novel insight for further studies on the effects of exercise in AD treatment.

## Ethics Statement

This study complied with the Animal Care and Institutional Ethical Guidelines in China. All animal experiments were under approval by the East China Normal University (certificate number: m20180108).

## Author Contributions

XZ, JB and BX designed the study. NZ, FL and XC carried out the animal experiments. TH and TL analyzed the experimental results. XZ, QH and TL wrote the first draft of the manuscript. QH contributed equally with XZ to this article and could be considered as common first author. TL contributed same to this article and could be considered as common corresponding author. All authors critically read and approved the final manuscript.

## Conflict of Interest Statement

The authors declare that the research was conducted in the absence of any commercial or financial relationships that could be construed as a potential conflict of interest.
